# Sex Assignment in Cases of Ambiguous Genitalia

**DOI:** 10.7759/cureus.74730

**Published:** 2024-11-29

**Authors:** Hajira Mohammed, Nesa Ansari, Ahmed Zafar Baig, Joud J Alshowaikhat, Madiha M Uz Zama, Jumana Hussain Timraz, Ruqayyah A Ahmed, Mohommad Samy

**Affiliations:** 1 Department of Medicine and Surgery, Batterjee Medical College for Science and Technology, Jeddah, SAU; 2 Department of General Surgery, General Medicine Practice Program, Batterjee Medical College for Science and Technology, Jeddah, SAU

**Keywords:** ambiguous genitalia, androgen receptor, external genitals, gonadal dysgenesis, sex assignment

## Abstract

Ambiguous genitalia is a rare disorder where it is unclear whether an infant’s external genitals are male or female. This can be attributed to various internal and external etiologies, such as androgen receptor abnormalities, gonadal abnormalities (such as gonadal dysgenesis or Klinefelter syndrome where a male has an extra X chromosome), enzymatic defects, etc. Correction of such atypical genitalia requires a multidisciplinary approach, including but not limited to surgeons and therapists. It is important to keep in mind that the treatment plan is tailored according to the specific etiology that is causative of the patient’s condition, along with the anticipated perioperative and postoperative complications. Islamically speaking, this topic has been addressed in various Islamic literature and disciplines, including guidelines for dealing with this medical condition. Moreover, follow-up of the patient must be done to allow smooth integration into society.

## Introduction and background

Ambiguous genitalia is a rare disorder where an infant’s external genitals do not appear to be evidently male or female. A  newborn  with ambiguous (also referred to as *atypical* or *intersex*) genitalia may have characteristics from both sexes, or its genitals might be inadequately developed [1]. To achieve the best possible long-term outcome, ambiguous genitalia in a baby is treated as a life-threatening situation that necessitates trained and specialized surgery in addition to distinctive studying of the case at a research center and specialized in-depth judgment [1].

An infant’s external genitalia is frequently evaluated to determine the sex. Infants with atypical (intersex) external genitalia may have internal or genetic sex organs that are different from their outward genitalia. This condition is also commonly referred to as a disorder of sex development (DSD) [1,2].

It can be hard for families and healthcare providers to care for  newborns  with ambiguous genitals. Medical, ethical, and social considerations must be considered while determining whether to classify them as male or female [2]. Through an analysis of the experiences of people with  ambiguous  genitalia, along with the opinions of medical professionals and ethicists, this study seeks to clarify matters related to policies and procedures when it comes to sex assignment.

Thus, this review article's primary  goal is to provide an overview of the ambiguous genitalia types, etiology, epidemiology, sex assignment criteria, cultural, ethical, and religious influences, patient perspectives, and future directions in Saudi Arabia.

## Review

Epidemiology

It is estimated that one in 4,500 babies are born with DSD globally. Depending on the region of the world, it may differ [3]. In Saudi Arabia, a study was done for 203 children with this condition between the ages of one day and 13 years. As a result, 139 (68.5%) were genetic females (46,XX). Congenital adrenal hyperplasia (CAH) was the most common cause in 137 patients, and only two patients were reported with clitoromegaly, while the remaining patients had androgen sensitivity, alpha reductase deficiency, mixed gonadal dysgenesis, hypogonadotrophin deficiency, or hypospadias, followed by some clinical manifestations of salt-wasting and some degree of virilization. Out of 203 patients, 64 (31.5%) were genetically male (46,XY). Among these, 20 (31.3%) had androgen insensitivity, 9 (14.1%) had 5-alpha reductase deficiency, and the remaining presented with mixed gonadal dysgenesis, hypospadias, or enzyme deficiencies [4].

In a previous research, 28 newborns with DSD were reported from a tertiary care institution, King Faisal Specialist Hospital and Research Centre in Riyadh, Saudi Arabia [3]. The incidence rate was calculated to be 0.4 per 1000 live births. Due to varying categorization standards and cultural effects, precise prevalence estimates are still difficult to determine. There are concerns that endogamous groups have a greater DSD prevalence as Western countries have low consanguinity. In contrast, countries with higher consanguinity, such as Saudi Arabia and other areas, may experience an increased prevalence of both 46,XY and 46,XX DSDs [3].

Genetics of sex differentiation and determination

The coordinated action of several genes and pathways is involved during the sequential process of sex development. This process leads to the development of differentiated internal sex organs, functional gonads, secondary sexual characteristics that externalize after puberty, and external genitalia. Sex determination and sex differentiation are two distinct processes of prenatal sex development. Approximately, after six to seven weeks of gestation, sex determination occurs, which is the initial step right after conception. Either testes or ovaries evolve from the undifferentiated gonads during this phase. Contrarily, sex differentiation occurs later and is dependent on hormones produced following the formation of gonads distinctive to a certain sex. These hormones play a critical role in regulating the dependent embryonic structures' continued growth, eventually leading to the development of the internal and external phenotypes of males and females [5].

Male-Specific Pathway 

The presence of Y chromosomes, particularly the SRY (sex-determining region Y) gene, is the initial step to determining the male sex and the beginning of a male-specific pathway [5]. 

The expression of the SRY gene in pre-Sertoli cells serves as a critical switch in the male-specific pathway, driving gonadal development toward the testes. The development of testis is activated by the SOX9 signaling pathway, which is then activated by SRY and NR5A1. Genes like GATA4, anti-Mullerian hormone, and NR0B1 are important for the early development of the testis. The growth of Leydig cells and the regression of Müllerian structures depend on AMH, which is released by Sertoli cells [5]. The androgens produced by the testes help in the development of the male external genitalia (penis, scrotum) and internal organs such as epididymis, vas deferens, and seminal vesicle. For the production of sex steroids, the steroidogenic pathway which begins with cholesterol is necessary. However, conditions such as CAH can interfere with steroidogenesis and impact the development of the fetal genitalia. Conditions like androgen insensitivity syndrome (AIS) and 5-alpha reductase deficiency are caused by disruptions in the male-specific pathway. Individuals with XY chromosomes in AIS, despite the production of testosterone, are unable to respond due to abnormalities in the androgen receptor (AR), which, in turn, results in female phenotype. In 5-alpha reductase deficiency, ambiguous or underdeveloped male genitalia is due to insufficient conversion of testosterone to DHT [5,6].

Female-Specific Pathway 

In the absence of the Y chromosome and SRY gene, the female-specific pathway operates, which enables the default development of female genitalia, and it involves suppression of SOX9. The production of estrogen and progesterone is by the bipotential gonads which develop into ovaries. In this pathway, the lack of AMH allows the Müllerian ducts to develop into the female reproductive system. CAH is a condition that disrupts the female pathway by excessive androgen production in genetic females (46,XX), which results in ambiguous genitalia and virilization. Increased adrenal androgens and adrenal hyperplasia are caused by enzyme deficiencies that affect cortisol synthesis [5,6].

The male- and female-specific pathways in the context of ambiguous genitalia involve distinct hormonal and genetic mechanisms that impact the development of external and internal genitalia. For diagnosing and managing conditions associated with ambiguous genitalia, it is very crucial to understand these pathways [5,6].

Etiology and types

In the realm of medical research on DSD or ambiguous genitalia, the classification of this complicated condition into its types provides a crucial framework for comprehending and addressing the various manifestations of this phenomenon. A 2019 study [7] categorized ambiguous genitalia or DSD into four groups based on their etiologies. Each of these types contributes uniquely to the wide range of clinical presentations, emphasizing the complex interaction between environmental, hormonal, and genetic influences throughout the formation of genital morphology. Having insight into the classification is important for delivering personalized treatment. The following section goes further into each kind, offering light on its unique traits, diagnostic approach, and potential management approaches for each.  The classification as per the aforementioned 2019 study is given next.

*AR* *Anomalies*

It includes the partial androgen insensitivity syndrome (PAIS) and complete androgen insensitivity syndrome (CAIS). In the case of PAIS, abnormalities in the AR gene result in different degrees of inadequate masculinization in those with XY chromosomes. Therefore, individuals may present with ambiguous genitalia that leans toward female or underdeveloped male characteristics, and treatment often involves assigning the gender that matches their most dominant physical traits. However, CAIS is caused by mutations that render the AR substantially resistant to the actions of androgens, resulting in a complete lack of masculinization in affected individuals despite having XY chromosomes. Hence, the diagnosis involves the female phenotype at birth with the presence of undescended testes in the lower abdomen or inguinal canal, with surgery considered to remove the undescended testes [8-12].

Gonadal Abnormalities 

The primary example of such abnormalities is gonadal dysgenesis (incomplete or abnormal), which is a disorder in which the development of the ovaries or testes fails, usually due to abnormalities in the sex-determining region of the Y chromosome (SRY). In cases of complete dysgenesis, individuals typically present as female, while in mixed gonadal dysgenesis, both male and female tissues may be present, which makes gender assignment more complicated in these patients. Treatment is based on the dominant genital appearance [13].

Second, true hermaphroditism, on the other hand, is a condition in which the patient has both ovarian and testicular tissue. The development of ovotestis includes a variety of genetic and hormonal variables. These cases are particularly complex because the individual may have mixed genitalia(46,XX/46,XY), requiring extensive evaluation to decide on gender assignment. In these cases, gender is usually assigned based on the most functional gonad and external genital appearance [13,14].

Finally, Klinefelter syndrome (47, XXY) and Turner syndrome (45,X) are genetic diseases that influence male and female development, respectively. Males with Klinefelter syndrome have an additional X chromosome, whereas females with Turner syndrome have a missing or partially missing X chromosome. These genetic anomalies interfere with normal physical and hormonal development in affected individuals and, therefore, lead to incomplete or abnormal development of reproductive organs resulting in ambiguous genitalia [15-19].

Enzyme Defects   

Ambiguous genitalia can also result from disturbances in the enzymatic pathways that are involved in the metabolism and production of steroid hormones. These enzyme deficits, whether caused by glandular or peripheral sources, play a critical role in normal sexual development. The most common enzymatic deficits are 46,XX/CAH, and 46XY/5-alpha reductase 2 (5-AR2) [20]. 

CAH is a hereditary condition characterized by cortisol synthesis enzyme abnormalities, most commonly caused by a lack of 21-hydroxylase. This causes elevated androgen levels, resulting in ambiguous genitalia in females and partially undeveloped genitalia in males. Females typically present with virilized (male-like) genitalia, though they are genetically female (46,XX). In these cases, treatment often involves stabilizing hormone levels and assigning the child a female gender. Although males with CAH do not usually present with ambiguous genitalia, they still need early diagnosis and management to prevent complications like a salt-wasting crisis, which can be life-threatening due to insufficient aldosterone which leads to electrolyte imbalances. Additionally, early monitoring is needed to address early puberty and eventually, fertility issues that may arise if hormonal imbalances are not corrected. Excess androgen production from these deficiencies leads to masculinization of external genitalia. Later in life, individuals of either sex may develop hypertension due to the deficiency of 11β -hydroxylase [20-23].

5-AR2 deficiency is a disease caused by mutations in the SRD5A2 gene that alter testosterone conversion to dihydrotestosterone (DHT). This results in ambiguous or undeveloped external genitalia at birth, despite being genetically male, due to a lack of DHT. At puberty, many experience masculinization due to a late surge of testosterone, so they are often assigned male at birth, though treatment should be guided by post-pubertal changes. Females with 5-AR2 deficiency may have female genitalia that are underdeveloped [20,21,22].

Exposure to Exogenous Factors 

During fetal development, the natural process of sexual differentiation can be disrupted by exogenous factors such as the presence of an androgen-producing tumor or maternal exposure to androgenic medicines. This disturbance can cause ambiguous genitalia in the fetus, irrespective of their genetic sex. Therefore in these cases, gender assignment is typically based on the appearance of external genitalia at birth, but further exploration and management are needed depending on the degree of genital ambiguity. However, these exogenous variables are fewer prevalent causes of ambiguous genitalia than the aforementioned genetic, enzymatic, and gonadal abnormalities. Nonetheless, they should be evaluated as potential contributory variables, particularly in cases when the root cause is not immediately evident [3].

To sum up, many different genetic, enzymatic, gonadal, and exogenous factors can cause ambiguous genitalia. Each of these factors has a unique etiology and phenotypic manifestation, which highlights the significance of a thorough assessment and tailored treatment.  

Sex assignment guidelines for management

As per the guidelines provided by the consensus statement published by the Lawson Wilkins Pediatric Endocrine Society (LWPES) and the European Society for Paediatric Endocrinology (ESPE), sex assignment of infants born with DSDs requires a multidisciplinary approach. This involves various specialists with an emphasis on individualized assessment by thorough diagnostic evaluation and active parent involvement, thus taking into account both medical and psychological factors [24]. It is necessary to follow the diagnostic procedures specific to causative etiology to facilitate proper and effective management. It is also advised to delay elective surgery till the child is capable of decision-making, thus ensuring the child’s rights and future autonomy are respected. Moreover, ethical considerations and psychosocial support are paramount, to assist the child in better understanding of its gender identity and to facilitate smooth integration into society [24,25].

Additionally, other experienced medical professionals have also suggested other factors to be evaluated during the gender assignment process, such as the patient’s chromosome structure, specific diagnosis, fertility, Prader stage, phallus length, and family wishes [26,27]. Specific guidelines for each disorder discussed are given next.

AR Anomalies

Stabilization comes first as a primary method for neonates. Ruling out CAH is very crucial for diagnosis, particularly the 21-hydroxylase deficiency variant, which can lead to life-threatening salt-wasting crises. Stabilizing electrolyte imbalance should be given immediate attention including management of hypoglycemia and administration of glucocorticoid therapy as needed. Hormonal therapy should be done to assess the levels of cortisol, androgens, and 17-hydroxyprogesterone to recognize the CAH and other various adrenal or androgen-related disorders. In this case, DHT and serum testosterone levels help identify the level of insensitivity [5,21,28].

Karyotype analysis should be done to determine the genetic sex (46,XX or 46,XY) for further investigations. Gonadal and pelvic ultrasound to determine the reproductive organs like testes, ovaries, and Mullerian structures would help identify if the disorder is caused by androgen insensitivity or other DSDs. Molecular testing for AR gene mutations for PAIS or CAIS confirms the mutations. Despite having adequate testosterone levels or DHT, hormonal assays may suggest androgen resistance, so molecular testing is very useful [5,28].

Delaying surgical or hormonal intervention in ambiguous genitalia is usually recommended after going through several diagnoses and family discussions to avoid premature decisions about gender assignment and surgery [5,28].

Reevaluation of androgen sensitivity at puberty by hormonal testing is a secondary method, as it is a critical timeline to reevaluate AR effectiveness in people with CAIS or PAIS. To evaluate how the body responds to rising androgen levels, hormonal assays like testosterone, DHT, FSH, and LH are repeated. Hormonal replacement therapy may be necessary if pubertal virilization may be insufficient in the case of PAIS. In CAIS, individuals will have female external genitalia, and during puberty may show primary amenorrhea. Genital ambiguity may worsen in partial AIS or may persist into puberty, and further intervention is necessary regarding the development of secondary sexual traits like body hair, gynecomastia, etc [28].

Long-term management often requires gonadectomy, especially in individuals with CAIS, to reduce the risk of gonadal tumors. This surgery is done after puberty so that the production of natural estrogen from the testes helps in pubertal development. Lifelong estrogen replacement is important to maintain the secondary female traits and bone health after going through gonadectomy. Testosterone replacement therapy may be considered if virilization is insufficient in cases of PAIS [28].

Gonadal Dysgenesis 

Primary methods for neonates require karyotyping and hormonal testing. In partial dysgenesis or abnormal dysgenesis, individuals may have XY karyotype and streak or nonfunctional gonads. Low levels of AHM, testosterone, or other sex hormones will be shown by hormonal assay. Individuals with 46,XY with digenetic gonads having SRY will have a higher risk of gonadal malignancy [28]. 

If individuals with 46,XY are raised as females, replacement therapy is required as a secondary method during puberty for the development of secondary sexual traits and the development of breasts. If they are raised as males, then testosterone is required [28,29,30].

True Hermaphrodite

To differentiate between ovarian, testicular, or mixed gonadal tissues, a gonadal biopsy is required as a primary method of identification. MRI or ultrasound to determine the gonads and the presence of Mullerian structures is performed. For secondary methods, surgical intervention and hormonal therapy are done depending on their assigned gender, like the administration of testosterone and estrogen for the development of external genitalia and gonads [5,11,28,31].

Turner and Klinefelter Syndromes

 In Klinefelter syndrome, neonates may not show signs at birth but features like macropains and low muscle tone (hypotonia) might be present. During puberty, boys will show delayed or incomplete development. Hence, testosterone replacement therapy is necessary to improve bone density, mood, voice deepening, muscle mass, and body hair [19,32].

In Turner syndrome, physical examination at birth shows swelling of hands and feet (lymphedema), a low set of ears, and a webbed neck. Immediate renal and cardiac assessment should be done as infants might have congenital heart defects in this syndrome, so neonates should go through an echocardiogram to check for aortic coarctation. Girls with this syndrome will be most likely to have growth failure. To improve height, growth hormone is initiated during early childhood. For secondary sexual traits and to start menstruation, girls will require estrogen therapy later to maintain the regularity of menstruation. Moreover, breast development would require progesterone therapy [5,17,18,32].

Common surgical procedures for ambiguous genitalia

Clitoral Reduction Surgery (Clitoroplasty)

This surgical method is designed for individuals with 46,XX CAH who experienced prenatal androgen exposure, resulting in an enlarged clitoris. The purpose of the procedure is to create a typical female genital appearance while preserving as much sensitivity as possible [29,33,34].

Vaginoplasty

This procedure is done to create a vaginal opening or enlarge the opening, as individuals with 46,XX and CAH may have absent or fused openings. Furthermore, it restores the opening for future menstrual flow [29,33,35].

Genital Reconstruction in 46,XY

This procedure is for individuals with 46,XY, with either AIS or PAIS. Ambiguity might be due to underdeveloped male external genitalia like hypospadias repair, which is a urethral opening that is under the penis. By this procedure, the urethral opening is repositioned on the tip of the penis, which allows the normal appearance of the genitalia and better urination [29,33,34].

Labiaplasty

This procedure is for individuals with CAH, where the labia is fused or masculinized. This surgical procedure restores the normal appearance of labia [33,34].

Gonadectomy

Individuals with 46,XY who have typically nonfunctional gonads with either gonadal dysgenesis or CAIS undergo gonadectomy, where there is an increased malignancy risk of undescended gonads. This surgical procedure is done after puberty to reduce the risk of tumors of the gonads. Moreover, to maintain the secondary sexual characteristics, hormonal replacement therapy is given postoperatively [35,36].

Ethical, cultural, and religious influence

Managing a patient with DSD is indeed difficult and complex. Among other things, biomedical, psychological, cultural, and religious components appear to have consequences.

Measures must be taken to improve counseling techniques and quality. It will be easier to communicate with patients and their families if the multidisciplinary team includes a religious scholar who is knowledgeable about genital ambiguity [37].

Another aspect of DSD is its vast range of clinical presentations. Another issue to take into account is the period between identification from the antenatal period into infancy or adolescence. The gender preference of a newborn or child with DSD can also be influenced by social, cultural, and economic factors in the attitudes of the parents. For both health care professionals and the parents, this situation can be very stressful, particularly if the expectations and the genetic, gonadal, and phenotypic sexual features are at clash. For individuals with atypical somatic sex developments, culture plays an important role in gender determination. The rejection of assigned gender or acceptance of it to psychosexual developments, and medical management, all have cultural influences [38].

In every society, neglect or exclusion can be directed against individuals or children with DSDs. For instance, people with ambiguous genitalia are frequently referred to negatively in Vietnam and India. These individuals are mistreated, feared, and dwelled on the margins of society [13].

This perspective Islamically is that in the Qur’an, the Almighty decrees that all human beings, whether they are male or female, are descended from Adam and Eve. Men and women have unique duties, obligations, and accountabilities due to differences in their physiology, anatomy, and psychology [37].

When it comes to establishing legal guidelines, for example, classical Islamic law acknowledges four genders in humans: male, female, DSD/intersex (khunsa), and the effeminate male (mukhannath). An individual with disorders of sex development DSD (khunsa) is described as “a person with both male and female organs or with an opening in place of a sexual organ” according to the Islamic scholars with somatic sex ambiguity due to DSD, 46,XX congenital hyperplasia or 46, XY androgen insensitivity [38].

In Islam, intersex due to DSD (khunsa) is accepted because the Prophet Muhammad (peace be upon him) is said to have answered in response to a question regarding how to identify the sex of a child born with two opposing sex organs. The determining factors include the organ from which the child urinates, the ability to produce sperm or become pregnant, or secondary sexual characteristics [38].

Intersex due to DSD (khunsa) has been divided into subcategories: non-problematic/discernible (khusna ghayr wadhih) and problematic/intractable (khunsa musykil).

A person having both male and female genitalia (khunsa ghayr musykil/wadhih) can be classified as either a certain sex or gender depending on which genital organ is more dominant than the other. This individual can be classified as male if, for instance, he develops facial hair, ejaculates semen, or urinates from the penis. However, this individual would be considered female if she grows breasts and starts menstruating [38].

In contrast, a problematic/intractable intersex (khunsa musykil) is a person who continues to urinate from both the penis and the vagina, making it difficult for them to be classified as either male or female.

It is important to note that the basis for this religious classification is a concept of anatomy that underlies both modern imaging methods and the current understanding of embryology.

Shariah law states that if medical professionals recommend it, surgery may be carried out in these DSD (khunsa musykil) cases to help determine the intersex person's (khunsa's) true sex. This will allow the person to be assigned a specific gender [38]. Patients with genital ambiguity and DSD continue to be complex and challenging. Medical professionals and Islamic scholars around the world certainly need to bridge the gap between them to ensure better care and better guidelines for sex ambiguity with in-depth research and decision-making [37,38].

Future directions

Having a child with ambiguous genitalia, its understanding, and the reaction, only recently received systemic study. The worry and concern of some parents and caregivers about their child’s potential future in terms of stigma and sexual dysfunction from facing loneliness and isolation are paramount. Surgical techniques, advances in genetics, and endocrinology most likely will play a crucial role in adapting to the needs, identities, preferences, and treatments of individuals. Prognosis and management have improved over time for the patients for whom it is not an option for prenatal diagnosis or treatment. The diagnosis of DSD in children with atypical genitalia usually results in consequential stress and abnormal parenting strategies [13,15,39].

An example is when a mother thinks that their child with ambiguous genitalia has not received correct genital surgery, or when they favor one sex over the other. Later during adolescence, mothers stress that their child is maturing *differently* despite getting an earlier surgery [40].

Additionally, according to some parents’ concerns, the surgery of their child with DSD will not completely fix them, but rather it does not last long. Instead, the families need to be educated about the differences in sex as well as the condition their child has. The confidence of parents in their abilities to raise their children by the given sex is necessary for normal psychosocial development [39]. For optimal quality of life, it is essential for parents, patients, and caregivers to be open and have clear communication [41]. Networking with other parents who have experience with children with ambiguous genitalia can be a valuable source and extremely important for accepting their child’s condition. The significance of early detection, appropriate gender assignment, timely intervention, and psychological counseling is important for mental and physical growth. Maintaining the highest level of consistency between the patient's given sex and gender identity is the primary goal of sex assignment. Continued research and developments in medical management are required to improve the quality of life and outcomes of patients with ambiguous genitalia. The next step after assigning the gender based on the karyotype is surgical intervention, hormone therapy till puberty or some people opt for no intervention at all. However, if any surgical intervention can delay a child with ambiguous genitalia without harming the newborn, it should be postponed until with understanding a personal consent can be given [41,42].

Furthermore, the attitude of society continues to change and evolve as time passes, and with awareness growing about the inclusivity of intersex traits, discrimination, and stigma are reduced. Educating society about ambiguous genitalia and advocacy efforts might lead to understanding and acceptance, nurturing conditions where these individuals can thrive without judgment, and with ongoing improvements in technologies, family planning, and individuals may be able to make choices about their respective futures. Eventually, with acceptance, inclusivity, and a healthcare system, individuals with DSD will thrive [41,43,44].

## Conclusions

In conclusion, ambiguous genitalia is a medical emergency of the utmost importance and should be managed with a multidisciplinary approach specific to the causative etiology, making sure all ethical boundaries are respected. Special importance should be given to the patient's postoperative care, both mental and physical, to facilitate their smooth integration into society.
